# Frequency of clinically isolated strains of oral *Candida* species at Kagoshima University Hospital, Japan, and their susceptibility to antifungal drugs in 2006–2007 and 2012–2013

**DOI:** 10.1186/1472-6831-14-14

**Published:** 2014-02-20

**Authors:** Yoshiaki Kamikawa, Youichirou Mori, Tomohiro Nagayama, Junichi Fujisaki, Daisuke Hirabayashi, Ryoichi Sakamoto, Tomofumi Hamada, Kazumasa Sugihara

**Affiliations:** 1 Department of Oral Surgery, Kagoshima University Medical and Dental Hospital, 8-35-1 Sakuragaoka, Kagoshima 890-8544, Japan; 2 Field of Maxillofacial Diagnostic and Surgical Science, Department of Oral and Maxillofacial Rehabilitation, Kagoshima University Graduate School of Medical and Dental Sciences, 8-35-1 Sakuragaoka, Kagoshima 890-8544, Japan

**Keywords:** *Candida glabrata*, *Candida albicans*, Clinically isolated strains, Minimal inhibitory concentration, Resistant strains

## Abstract

**Background:**

The isolation frequency and susceptibility to antifungal agents of oral *Candida* isolates from patients with oral candidiasis (OC) were compared between studies conducted in 2006–2007 and 2012–2013.

**Methods:**

A total158 strains was isolated from 112 patients who visited Kagoshima University Hospital for the treatment of OC during the 14-month period from February 2012 and March 2013, and evaluated on the isolation frequency of each *Candida* strain and the susceptibility against antifungal drugs as compared to those evaluated in 2006–2007.

**Results:**

There was a higher frequency of xerostomia as a chief complaint and of autoimmune disease in the 2012–2013 study than in the 2006–2007 study. More than 95% of *Candida* isolates were *C. albicans* and *C. glabrata*. In addition, the proportion of the latter increased from 12.3% in the 2006–2007 study to 23.4% in the 2012–2013 study, while the proportion of the former decreased from 86.2% to 72.8%, respectively. *C. albicans* was isolated in almost all patients, while *C. glabrata* was only isolated concomitantly with *C. albicans*. Minimal inhibitory concentrations (MICs) were not significantly different between groups with a few exceptions. *Candida* isolates, of which MICs surpassed break points, apparently increased for miconazole and itraconazole against *C. glabrata* in the 2012–2013 study, but this was not statistically significant. As a result, more cases of autoimmune disease, a greater number of *C. glabrata* isolates, and higher resistance to azoles were seen in the 2012–2013 study than in the 2006–2007 study.

**Conclusion:**

These data indicate that with recent increases in *C. glabrata* infection, a causative fungus of OC, and in *C. glabrata* resistance to azoles, caution is needed in the selection of antifungal drugs for the treatment of OC.

## Background

In the early 1990s, *Candida albicans* resistant to fluconazole was reported to be frequently isolated from the oral cavity of acquired immunodeficiency syndrome patients. An epidemiological study was conducted on the susceptibility to antifungal drugs of *Candida* strains isolated from patients with invasive candidiasis [1]. Global surveillance programs have been implemented for dealing with candidal resistance to antifungal drugs, such as the SENTRY program on fungi resistant to amphotericin B in 2006–2007 [2] and reports on candidal susceptibility to candin-type antifungals [3-6]. There are 2 reports on domestic surveillance in Japan: Surveillance 99 by the Japan Invasive Mycosis Surveillance Study Group (JMS) [7] and Report by Japan Antifungal Surveillance Program (JASP) [8-10]. However, these surveillance programs focused on strains isolated from clinical cases of deep-seated mycosis, and only 1 report, our previous study performed in 2006–2007, was conducted on oral candidiasis [11].

The isolation frequency of *Candida* strains is likely to change over time, as shown by the comparisons of previous studies conducted in different time periods. For example, *C. glabrata* was rarely isolated from AIDS patients with oral candidiasis (OC) or esophageal candidiasis in Sudo’s study published in 1997 [12]; in contrast, *C. glabrata* was detected in 12.3% of patients with OC in our study, in which itraconazole (ITCZ) was effective despite the existence of resistant *C. glabrata* against ITCZ [11]. In addition, while resistant *C. glabrata* against ITCZ occurred with high frequencies of 50% and 72.7% in OC and non-OC patients of this study, respectively, [11], a previous Japanese surveillance study demonstrated resistant *C. glabrata* in only 1.0–9.3% of patients with mycosis, mainly deep-seated mycosis [7-10]. Thus, the isolation frequency of *Candida* species and their resistant strains varies over time, but no study has reported variations in frequency of OC over time in the same health facility.

The present study was undertaken to evaluate antifungal drugs against *Candida* strains isolated form the oral cavity of OC patients who visited Kagoshima University Hospital in 2012–2013 (14 months) and to assess the isolation frequency of *Candida* species and their resistant strains in comparison with our previous study conducted in 2006–2007 (24 months) in order investigate the use antifungal drugs in the treatment of OC.

## Methods

### Subjects

This study analyzed 158 strains isolated from 112 patients who visited the Department of Oral Surgery, Kagoshima University Hospital, for the treatment of OC during the 14-month period from February 2012 and March 2013. Strains collected from patients having received antifungal drug therapy within 3 months from collection were excluded from this study.

### Analysis of background variables of *Candida*-isolated patients

An analysis was conducted for age, gender, underlying systemic disease, steroid hormone use, denture use, chief complaints, and oral disease in the 112 patients from whom *Candida* was isolated.

### Collection of *Candida* from the oral cavity and bacterial culture

Prior to the diagnosis of OC, the affected area of the oral cavity was wiped with a sterile cotton swab pre-immersed in sterile physiological saline. Fluid sample collected on the swab was smeared onto a CHROMagar *Candida* Culture Medium (Nippon Becton and Dickinson Company, Ltd.) and incubated for 48 hours at 36°C.

### Identification of *Candida* species

Positive culture of *Candida* species was determined by colorimetric observation of colonies in the CHROMagar *Candida* culture medium containing synthetic colorimetric substrates which are changed to be chromogenic by enzymes contained in *Candida* species. Confirmation of the determination of Candida species was then done by PCR based on the method reported from Kanbe et al., briefly fungal cells grown in the CHROMagar Candida culture medium were harvested by centrifugation, washed three times with distilled water, and the DNAs were extracted and purified according to the protocol of a DNA purification kit, Fast DNA Kit (BP Biomedical, U.S.). Then, the DNA samples were amplified by PCR and the PCR products were analyzed by agarose gel electrophoresis to determine the kind of Candida species [13].

### Diagnosis of OC

Fluid collected on the swab from the affected area was transferred onto a glass slide for Gram staining by a routine method. A diagnosis of OC was made when colony formation was noted during incubation and the presence of pseudo mycelium was shown by Gram staining [14].

### Evaluation of susceptibility to antifungal drugs

*Candida* strains detected by the above-mentioned methods were re-seeded onto Sabouraud agar medium (Nippon Becton and Dickinson Company, Ltd.), followed by 48-hour incubation at 36°C. Then the culture was diluted and adjusted to a McFarland factor of 0.5 by measuring the optical density at 530 nm using a spectrophotometer ViSpec II (Kyokuto, Japan), and applied for the assay to determine the susceptibility. A minimum inhibitory concentration (MIC)of each of AMPH-B, 5-FC, FLCZ, MCZ, ITCZ, and MCFG against these *Candida* strains were determined with a Yeast-like Fungal Sensitivity Kit ASTY (Kyokuto Pharmaceutical) compatible with the National Committee for Clinical Laboratory Standards (NCCLS) M-27A method [15]. The kit includes 11 concentrations of each antifungal drug in a 96-well microplate, i.e. final concentrations 0, 0.03, 0.06, 0.125, 0.25, 0.5, 1, 2, 4, 8, and 16 μg/mL for amphotericin B, miconazole, and micafungin, 0, 0.125, 0.25, 0.5, 1, 2, 4, 8, 16, 32, 64 μg/mL for fluconazole and 5-FC, and 0, 0.015, 0.03, 0.06, 0.125, 0.25, 0.5, 1, 2, 4, and 8 μg/mL for itraconazole and micafungin. The concentration “0 μg/mL” is regarded as a control. Strains with high MIC were subjected to re-measurement of MIC with a Yeast-like Fungal DP Eiken (Eiken Chemical) compatible with the Clinical and Laboratory Standards Institute (CLSI) M27-A3 method [10,16]. MIC50 and MIC90, MICs required to inhibit the growth of respective 50% and 90% of *Candida* species, were calculated. Resistant strains were determined by the following MIC criteria: AMPH-B, FLCZ, ≥64 μg/mL; 5-FC, ≥32 μg/mL; MCZ, ≥4 μg/mL; MCFG, >2 μg/mL; and ITCZ, ≥1 μg/mL.

### Informed consent and ethical guideline

All enrolled patients provided written informed consent upon directly providing verbal and written detailed explanation of this investigation. This study was approved by the Human Ethics Committee of the Kagoshima University Medical and Dental Hospital (No.23-18 and 23–27).

### Statistical analysis

Fisher’s exact probability test was employed to determine differences in gender (2 × 2), chief complaints (2 × 8), systemic disease (yes or no; 2 × 2), steroid use (2 × 2), presence of dentures (2 × 2), oral candidiasis (2 × 4), composition of isolated *Candida* species (2 × 7), and the incidence of resistant strains between the 2006–2007 study and the 2012–2013 study. The difference in age between the 2 studies was examined by Student’s *t*-test. P value less than 0.05 was regarded as statistically significant.

## Results

### Background variables

As shown in Table 1, there were no statistical differences in background variables in terms of gender, age, use of steroid hormone treatment, presence and the kind of systemic diseases and presence of dentures between patients in the 2006–2007 study and those of the 2012–2013 study. Among patients with 1 chief complaint, xerostomia was higher in the 2012–2013 study (23.2%) than in the 2006–2007 study (8.8%), while tylosis linguae was lower at 7.0% and 0%, respectively. Collectively, the composition of chief complaints was significantly different between the 2006–2007 patients and 2012–2013 patients as analyzed by Fisher’s exact probability test (p < 0.05). Despite no significant difference in the occurrence of systemic disease between the 2 studies (77.2% vs. 86.6%), the presence of autoimmune diseases was higher in the 2012–2013 study than in the 2006–2007 study (27.6% vs. 6.7%, respectively).

**Table 1:** Background variables of patients with oral candidiasis enrolled in the 2006–2007 and 2012–2013 studies

		**2006-2007**		**2012-2013**		**Test**	
**Subjects number**	**Total**	**57**		**112**			
Gender	Male	19	(33.3)	30	(26.8)	Fisher’s exact probability test	
	Female	38	(66.7)	82	(73.2)	[2 × 2]	N.S.
Age	Average [max-min]	67.9	[26–90]	69.6	[21–97]	Student’s t-test	N.S.
Chief Complaints	Heat sensation(HS)	23	(40.4)	46	(41.1)		
	Taste abnormality (TA)	2	(3.5)	0	(0)		
	Xerostomia (X)	5	(8.8)	26	(23.2)		
	Tylosis linguae (TL)	4	(7.0)	0	(0)	Fisher’s exact probability test	
	Rubor/Erosion (R/E)	4	(7.0)	8	(7.1)	[2 × 8]	
	HS + TA + X	9	(15.8)	16	(14.3)	p < 0.05	
	HS + TA + R/E	5	(8.8)	8	(7.1)		
	HS + TL	5	(8.8)	8	(7.1)		
	Total	57	(100)	112	(100)		
Systemic disease	Yes	44	(77.2)	97	(86.6)	Fisher’s exact probability test	
	None	13	(28.8)	15	(13.4)	[2 × 2]	
	Total	57	(100)	112	(100)	N.S.	
	Endocrine	22	(29.3)	22	(17.3)		
	Cardiovascular	18	(24.0)	28	(22.0)		
	Gastrointestinal	123	(16.0)	12	(9.4)	Fisher’s exact probability test	
	Malignant tumor	8	(10.7)	12	(9.4)	[2 × 7]	
	CNS	4	(5.3)	10	(7.9)	N.S.	
	Viral	6	(8.0)	8	(6.3)		
	Autoimmune	5	(6.7)	35	(27.6)		
	Total	75	(100)	127	(100)		
Steroid	Dose	12	(21.1)	28	(25.0)	Fisher’s exact probability test	
	Non-dosed	45	(78.9)	84	(75.0)	[2 × 2]	
	Total	57	(100)	112	(100)	N.S.	
Denture	Wearer	37	(64.9)	57	(50.9)	Fisher’s exact probability test	
	Non-wearer	20	(35.1)	55	(49.1)	[2 × 2]	
	Total	75	(100)	112	(100)	N.S.	
Oral candidiasis	Pseudomembranous	42	(73.7)	42	(37.5)		
	Erythematous	11	(19.3)	52	(46.4)	Fisher’s exact probability test	
	Hypertrophic	2	(3.5)	4	(3.6)	[2 × 2]	
	Mixed	2	(3.5)	14	(12.5)	N.S.	
	Total	55	(100)	112	(100)		

Figure in parentheses represents the percentage except the age.

### Classification of oral candidiasis (OC)

Classification of diagnosed OC cases is also shown in Table 1. Among the 57 patients in the 2006–2007 study, OC was most frequently classified as pseudomembraneous OC (73.7%), followed by erythematous OC (19.3%). In the 2012–2013 study, however, erythematous OC accounted for half (46.4%) of OC cases, followed by pseudomembranous OC (37.5%) and mixed OC (erythematous and pseudomembranous, 12.5%), but no statistical difference was noted.

### Isolated *Candida* species

In total, 158 strains of *Candida* were isolated in patients in the 2012–2013 study. The most frequently isolated *Candida* species were *Candida albicans* (*C. albicans*, 116 strains, 73.4%), followed by *C. glabrata* (36 strains, 22.8%), *C. tropicalis* (3 strains, 1.9%), *C. parapsilosis* (2 strains, 1.3%), and *C. krusei* (1 strain, 0.6%) (Table 2). When analyzed on a patient basis, *C. albicans* was isolated in all patients (n = 112), while *C. glabrata* was never found on its own, only concomitantly with *C. albicans* in 36 patients. More than one kind of *Candida* species was isolated in 41 patients, with concomitant *C. albicans* and *C. glabrata* being the most common combination in approximately 90% of these patients. In contrast, among 65 strains of *Candida* isolated in patients in the 2006–2007 study, 56 strains of *C. albicans* (86.2%), 8 strains of *C. glabrata* (12.3%), and 1 strain of *C. tropicalis* (1.5%) were identified, and no strains of *C. parapsilosis* and *C. krusei* were detected. Thus, in the 2012–2013 study, the number of isolated strains of *C. glabrata* was almost double that of the 2006–2007 study, and the ratio of *C. albicans* to all *Candida* strains was therefore significantly lower in the 2012–2013 study when analyzed on a strain-basis or on a patient-basis. Thus, in both patient-basis and strain-basis analyses, a statistical difference was observed between the 2006–2007 study and the 2012–2013 study (p < 0.01).

**Table 2:** **Distribution of *Candida *** **species isolated from patients with oral candidiasis in the 2006–2007 and 2012–2013 studies**

***Candida *** **species**	**Number of patients (%)**				**Test**
	**2006-2007**		**2012-2013**		
Single species isolated					
*Candida albicans* (CA)	49	(86.0)	71	(63.4)	
*Candida glabrata* (CG)	1	(1.8)	0	(0)	
*Candida tropicalis* (CT)	0	(0)	0	(0)	
*Candida parapsilosis* (CP)	0	(0)	0	(0)	
*Candida krusei* (CK)	0	(0)	0	(0)	Fisher’s exact probability test
Plural species isolated					[2 × 10]
CA + CG	6	(10.5)	36	(32.1)	p < 0.01
CA + CT	0	(0)	3	(2.7)	
CA + CP	0	(0)	1	(0.9)	
CA + CG + CT	1	(1.8)	0	(0)	
CA + CP + CK	0	(0)	1	(0.9)	
Total	57	(100)	112	(100)	
***Candida * species**	**Total number of *Candida *** **strains isolated (%)**				**Test**
	**2006-2007**		**2012-2013**		
*Candida albicans*	56	(86.2)	112	(72.8)	
*Candida glabrata*	8	(12.3)	36	(23.4)	
*Candida tropicalis*	1	(1.5)	3	(1.9)	Fisher’s exact probability test
*Candida parapsilosis*	0	(0)	2	(1.3)	[2 × 5]
*Candida krusei*	0	(0)	1	(0.6)	p < 0.01
Total	65	(100)	154	(100	

### Susceptibility to antifungal drugs

Since few (1 to 3 strains) of the *Candida* species other than *C. albicans* and *C. glabrata* were isolated in either study, MIC values of antifungal drugs against *C. tropicalis*, *C. parapsilosis,* and *C. krusei*, which were never isolated alone but only in combination with *C. albicans* and *C. glabrata*, were not reliable. Therefore, the results of susceptibility testing mainly refer to the MICs of *C. albicans* and *C. glabrata* (Table 3).

**Table 3:** **Antifungal susceptibility profile, against amphotericin B, flucytosine, fluconazole, miconazole, itraconazole, and micafungin, of *Candida *** **species isolated from patients with oral candidiasis in the 2006–2007 and 2012–2013 studies**

**a. amphotericin B (AMPH-B)**																
**Year**	**Strain**	**No. of strains**	**MIC (μg/mL)**												**MIC**_**50**_	**MIC**_**90**_
			**<0.03**	**0.03**	**0.06**	**0.125**	**0.25**	**0.5**	**1**	**2**	**4**	**8**	**16**	**>16**		
2006-2007	*C. albicans*	56					8	43	3					2	0.5	0.5
	*C. glabrata*	8				1	2	2	2	1					0.5	2
	*C. tropicalis*	1					1								0.25	0.25
2012-2013	*C. albicans*	112				4	12	86	9	1					0.5	1
	*C. glabrata*	36				1	2	16	14		2			1	0.5	1
	*C. tropicalis*	3						1	2						1	1
	*C. parapsilosis*	2	2												<0.03	<0.03
	*C. krusei*	1						1							0.5	0.5
**b. flucytosine (5-FC)**																
**Year**	**Strain**	**No. of strains**	**MIC (μg/mL)**												**MIC**_**50**_	**MIC**_**90**_
			**<0.125**	**0.125**	**0.25**	**0.5**	**1**	**2**	**4**	**8**	**16**	**32**	**64**	**>64**		
2006-2007	*C. albicans*	56	18	8	24	3	1							2	0.25	0.5
	*C. glabrata*	8	4	1		1					2				<0.125	16
	*C. tropicalis*	1		1											0.125	0.125
2012-2013	*C. albicans*	112	56	1	43	3	3	4					1	1	0.25	1
	*C. glabrata*	36	20			6	6			2	1		1		<0.125	8
	*C. tropicalis*	3	3												<0.125	<0.125
	*C. parapsilosis*	2													2	2
	*C. krusei*	1			1			1							0.25	0.25
**c. fluconazole (FLCZ)**																
**Year**	**Strain**	**No. of strains**	**MIC (μg/mL)**												**MIC**_**50**_	**MIC**_**90**_
			**<0.125**	**0.125**	**0.25**	**0.5**	**1**	**2**	**4**	**8**	**16**	**32**	**64**	**>64**		
2006-2007	*C. albicans*	56			22	22	5	1	1	1		1		3	0.5	4
	*C. glabrata*	8			1	2		2	1		1			1	2	>64
	*C. tropicalis*	1												1	>64	>64
2012-2013	*C. albicans*	112	4		33	51		2	1	3	3			3	0.5	2
	*C. glabrata*	36						4	1	5	6	7	7	2	16	64
	*C. tropicalis*	3						1				2			32	32
	*C. parapsilosis*	2						2							2	2
	*C. krusei*	1						1							2	2
**d. miconazole (MCZ)**																
**Year**	**Strain**	**No. of strains**	**MIC (μg/mL)**												**MIC**_**50**_	**MIC**_**90**_
			**<0.03**	**0.03**	**0.06**	**0.125**	**0.25**	**0.5**	**1**	**2**	**4**	**8**	**16**	**>16**		
2006-2007	*C. albicans*	56		2	22	20	8	1						3	0.125	0.25
	*C. glabrata*	8			1		2		2	1	2				1	4
	*C. tropicalis*	1													0.125	0.125
2012-2013	*C. albicans*	112			2	20	64	18	1	1	1	2		3	0.25	0.5
	*C. glabrata*	36				1		5	12	7	3	3	2	3	2	16
	*C. tropicalis*	3						1	1		1				1	4
	*C. parapsilosis*	2							1	1					1-2	2
	*C. kurusei*	1									1				4	4
**e. itraconazole (ITCZ)**																
**Year**	**Strain**	**No. of strains**	**MIC (μg/mL)**												**MIC**_**50**_	**MIC**_**90**_
			**<0.015**	**0.015**	**0.03**	**0.06**	**0.125**	**0.25**	**0.5**	**1**	**2**	**4**	**8**	**>8**		
2006-2007	*C. albicans*	56		4	20	14	9	2	1	3		1		2	0.06	1
	*C. glabrata*	8			1		1	1	1	1	1			2	0.05	>8
	*C. tropicalis*	1													>8	>8
2012-2013	*C. albicans*	112	2		12	34	50	5	4	1	1			3	0.125	0.25
	*C. glabrata*	36				1	3	3	5	9	8	2		5	1	>8
	*C. tropicalis*	3							1	1	1				1	2
	*C. parapsilosis*	2					1	1							0.25	0.25
	*C. kurusei*	1							1						0.5	0.5
**f. micafungin (MCFG)**																
**Year**	**Strain**	**No.of strains**	**MIC (μg/mL)**												**MIC**_**50**_	**MIC**_**90**_
			**<0.03**	**0.03**	**0.06**	**0.125**	**0.25**	**0.5**	**1**	**2**	**4**	**8**	**16**	**>16**		
2006-2007	*C. albicans*	56	32	9	13					1				1	<0.03	0.06
	*C. glabrata*	8	1		2	2	1	1						1	0.125	>16
	*C. tropicalis*	1			1										0.06	0.06
2012-2013	*C. albicans*	112	95		12	2	1	1	1						<0.03	0.06
	*C. glabrata*	36	16	1	1	3	5	6	1	2	1				0.125	1
	*C. tropicalis*	3	2		1										<0.03	00.6
	*C. parapsilosis*	2	2												<0.03	<0.03
	*C. kurusei*	1													0.5	0.5

#### Amphotericin B (AMPH-B)

The MIC was found to be low (≤2 μg/mL) in 63 (96.9%) of the 65 strains in the 2006–2007 study and in 153 (99.4%) of the 154 strains in the 2012–2013 study. Two strains of *C. albicans* in the 2006–2007 study and 1 strain of *C. glabrata* in the 2012–2013 study demonstrated high MIC of over 16 μg/mL. However, the presence of resistant strains could not be detected in the concentration range used in this analysis. There was no obvious difference in the susceptibility of the 2 main *Candida* species, *C. albicans* and *C. glabrata*, between the 2 studies, with MIC_90_ values of 0.5 μg/mL and 2.0 μg/mL, respectively, in the 2006–2007 study and of 1.0 μg/mL and 1.0 μg/mL, respectively, in the 2012–2013 study.

#### Flucytosine (5-FC)

Two strains of *C. albicans* in the 2006–2007 study and 2 strains of *C. albicans* and 1 strain of *C. glabrata* in the 2012–2013 study demonstrated high MIC of more than 32 μg/mL. There was no obvious difference in the susceptibility of *C. albicans* and *C. glabrata* between studies, with the 2006–2007 study demonstrating MIC_90_ of 0.5 μg/mL and 16 μg/mL, respectively, and the 2012–2013 study, of 1.0 μg/mL and 8.0 μg/mL, respectively.

#### Fluconazole (FLCZ)

MIC values were found below 64 μg/mL in 60 (92.3%) of the 65 strains in the 2006–2007 study and in 146 (92.4%) of the 158 strains in the 2012–2013 study. High MIC ≥64 μg/mL was recorded in 5 strains (9.5%) in the 2006–2007 study, of which 3 strains were *C. albicans*, 1 strain was *C. glabrata,* and 1 strain was *C. tropicalis*, and in 12 strains (7.6%) in the 2012–2013 study, of which 3 strains were *C. albicans* and 9 strains were *C. glabrata*, and which were all found to be FLCZ-resistant strains. A higher proportion of resistant *C. glabrata* strains were found in the 2012–2013 study (9/36, 25.0%) than in the 2006–2007 study (1/8, 12.5%). An increase in MIC_50_ of *C. glabrata* from 2 μg/mL in 2006–2007 to 16 μg/mL in 2012–2013 was also observed.

#### Miconazole (MCZ)

The MIC of 60 (92.3%) of the 65 strains in the 2006–2007 study and in 139 (88.0%) of the 158 strains in the 2012–2013 study were below 4 μg/mL. High MIC (≥4 μg/mL) was recorded for 3 strains of *C. albicans* (4.6%) and 2 strains of *C. glabrata* (25.0%) in 2006–2007, and for 19 strains (12.0%) in 2012–2013, of which 6 strains were *C. albicans* (5.4%), 11 strains were *C. glabrata* (30.6%), 1 strain was *C. tropicalis* (33.3%), and 1 strain was *C. krusei* (100%). Although MIC_90_ against *C. glabrata* in the 2012–2013 study (16 μg/mL) was higher than that in the 2006–2007 study (4.0 μg/mL), no other significant differences were seen between studies. The incidence of resistant *C. glabrata* was similar between the 2 studies at 25.0% (2/8 strains) and 30.6% (11/36 strains), respectively.

#### Itraconazole (ITCZ)

The MIC of 54 (83.1%) of the 65 strains in the 2006–2007 study and of 127 (80.4%) of the 158 strains in the 2012–2013 study were less than 1 μg/mL. High MIC (≥1 μg/mL) was recorded in 11 strains (16.9%) in 2006–2007, of which 6 strains were *C. albicans* (10.7%), 4 strains were *C. glabrata* (50.0%), and 1 strain was *C. tropicalis* (100%), and in 31 strains (19.6%) in 2012–2013, of which 5 strains were *C. albicans* (4.5%), 24 strains were *C. glabrata* (66.7%), and 2 strains were *C. tropicalis* (66.7%). There was a higher proportion of resistant strains of *C. glabrata* against ITCZ in the 2012–2013 study (24/36 strains, 66.7%) than in the 2006–2007 study (4/8 strains, 50.0%), but the difference was not statistically different. The MIC_90_ against *C. glabrata* was similar in both studies at >8.0 μg/mL, but that against *C. albicans* in the 2012–2013 study (0.25 μg/mL) was lower than that in the 2006–2007 study (1.0 μg/mL).

#### Micafungin (MCFG)

The MIC of 63 (96.9%) of the 65 strains in the 2006–2007 study and of 157 (99.4%) of the 158 strains in the 2012–2013 study were >4 μg/mL. High MIC (≥4 μg/mL) was recorded in only 2 strains (4.6%) in the 2006–2007 study, of which 1 strain each was *C. albicans* and *C. glabrata*, and 1 strain of *C. glabrata* in the 2012–2013 study. Although the MIC_90_ against *C. glabrata* in 2012–2013 (1.0 μg/mL) was lower than in 2006–2007 (>16 μg/mL), there was no other obvious differences in the susceptibility of *C. albicans* and *C. glabrata* between studies.

## Discussion

Non-*C. albicans Candida* (NCAC) strains have been isolated in increasing numbers in medically compromised patients including those with OC and denture stomatitis. *C. albicans* remains the most common fungus associated with infection, followed by *C. glabrata*. In clinically isolated samples from the blood, urine, and tracheal secretion of candidemia patients, the isolation frequency of *C. glabrata* has been found to be 16–26%, which is the highest among NCAC strains [17-20]. In the Prospective Antifungal Therapy Alliance (PATH Alliance®), in which 3,648 patients with candidemia were enrolled from 2004 to 2008 in the US, the most common *Candida* species was *C. albicans* (42.1%), followed by *C. glabrata* (26.7%), and the proportion of candidemia caused by NCAC strains was higher than that caused by *C. albicans*[21]. Such a high emergence of NCAC strains, especially of *C. glabrata*, in candidemia has recently been reported, with a higher incidence of *C. glabrata* found from 2006 to 2010 (range: 4.8–23.5%, P = 0.024) in Brazil [22]. In addition, the prevalence of infection due to a specific *Candida* species may vary by geographic region [23].

However, few reports exist on differences in variation of isolation frequency and its relation to candidemia in oral candidiasis. In contrast, there are several reports on oropharyngeal *Candida* infections due to *C. glabrata*[24,25]. In the present study, the isolation frequency of *C. glabrata* increased from 14.1% in 2006–2007 to 32.1% in 2012–2013 when assessed by the number of patients, indicating a similar tendency observed in Japanese OC patients. The tendency of a shift of NCAC species would also be attributable to frequent and wide use of azole antifungal drugs, such as fluconazole, in the treatment of mycosis [26]. Moreover, the isolation frequency of NCAC strains, especially *C. glabrata*, was reported to increase [26-28]. In Japan, as a result of a wide use of azole antifungal drugs as the standard remedy for deep-seated mycosis, it is supposed that the isolation frequency of *C. glabrata* became high.

By comparing background data of OC patients in the 2012–2013 study and in the 2006–2007 study, the following differences were observed: (1) the frequency of xerostomia as a chief complaint increased from 24.6% to 37.5% of patients, (2) autoimmune disorders as a systemic disease increased from 6.7% to 27.6%, and (3) erythematous OC as a type of OC increased from 19.3% to 46.4%. Differences in the frequency of xerostomia and autoimmune disorders were associated with an increase in patients with Sjögren’s syndrome, a chronic and progressive autoimmune disorder mainly characterized by xerostomia. Differences in erythematous OC may be partly attributed to an increase in simultaneous identification of *C. albicans* and *C. glabrata* as observed in 32.1% of patients in the 2012–2013 study. In fact, cases of mixed infection suggest that *C. albicans* enhances the invasiveness of *C. glabrata*, and leads to increased lactate dehydrogenase (LDH) release from a reconstituted human oral epithelium (RHOE) *in vitro*[29]. Furthermore, LDH release paralleled the observed histological damages in RHOE, which may be associated to the increase in erythematous OC.

By comparing the susceptibilities of patients in the 2012–2013 study with that of the 2006–2007 study, azole antifungal drugs (FLCZ, MCZ, and ITCZ) demonstrated high MIC_90_ values against *C. glabrata* in both studies. In particular, the MIC_90_ value of MCZ against *C. glabrata* increased from 4 μg/mL (2006–2007) to 16 μg/mL (2012–2013). Such high MIC values of azole antifungal drugs, which suggest a decrease in the susceptibility of *C. glabrata* to these drugs, have been recognized in several studies [17-19,30]. Decreased susceptibility to FLCZ was mainly seen against *C. glabrata* isolated from patients with blood stream infections, with a dose-dependent susceptibility rate of 76.5%, while the susceptibility rates of FLCZ were 100% for *C. albicans* and *C. parapsilosis*[19]. Similar results were also found for systemic candidiasis [18,20,23]. Decreases in the susceptibility of NCAC, especially of *C. glabrata*, resulted from resistance to FLCZ, leading to oropharyngeal *Candida* infections, which are more severe and more difficult to treat in HIV-infected and cancer patients [30]. The development of resistance in *C. glabrata* was observed against other azoles, such as ITCZ and ketoconazole [20,31]. In addition, in the present study, although statistical significance was not obtained, resistant strains of *C. glabrata* increased from 50.0% (4/8) in the 2006–2007 study to 66.7% (24/36) in the 2012–2013 study. In cases of other azole antifungal drugs, FLCZ and MCZ, resistant strains of *C. glabrata* increased slightly from 12.5% to 25.0% and from 25.0% to 30.6%, respectively. Thus, a tendency for increased isolation frequencies of resistant *C. glabrata* against azole antifungal drugs, especially ITCZ have been observed. Frequent exposure of antifungal drugs, especially azoles, is known to lead to an increase in resistant *Candida* strains, which has been reported since fluconazole was clinically available in 1989. After that new azole antifungal drugs, such as itraconazole and voliconazole, have been widely used for deep-seated mycosis. Thus, increases in clinically available antifungal azole drugs and in their clinical usages for the treatment of deep-seated mycosis could lead to an increment of resistant *Candida* strains. This tendency would be still continuing during the period of 2006–2013.

## Conclusions

In oral *Candida* species isolated from OC patients, the ratio of *C. glabrata* to *C. albicans* increased in the 2012–2013 study as compared to the 2006–2007 study, accompanying the tendency of increases in tolerant strains of *C. glabrata* against azole antifungal drugs and in cases of autoimmune disease. The higher isolation frequency of *C. glabrata*, especially of co-isolation frequency with *C. albicans*, may be associated with the increase in erythematous OC. Considering the increase in *C. glabrata* resistant strains against azoles, selection of appropriate antifungal drugs and dosing regimens against resistant *C. glabrata,* as well as against *C. albicans* is important.

## Competing interests

The authors declare that they have no competing interests.

## Author’s contribution

KY and MY participated in the design of the study, conducted the study, and conducted the data collection and analyses, and drafted the manuscript. NT, FJ, HD, SR, HT, and SK, participated in the design of the study and carried out the data collection. All authors contributed to the writing of the manuscript and critically reviewed the final version. All authors read and approved the final manuscript.

## Pre-publication history

The pre-publication history for this paper can be accessed here:

http://www.biomedcentral.com/1472-6831/14/14/prepub
